# Zwitterionic 1-{(*E*)-[(2-methyl­phen­yl)iminium­yl]meth­yl}naphthalen-2-olate

**DOI:** 10.1107/S1600536814008794

**Published:** 2014-05-17

**Authors:** Ammar Khelifa Baghdouche, Salima Mosbah, Youghourta Belhocine, Leïla Bencharif

**Affiliations:** a Laboratoire de Chimie des Matériaux, Faculté des Sciences Exactes, Département de Chimie, Université Constantine 1, Algeria

## Abstract

The title Schiff base, C_18_H_15_NO, crystallizes in its zwitterionic form and an N—H⋯O hydrogen bond closes an *S*(6) ring. The dihedral angle between the aromatic ring systems is 36.91 (10)°. Weak aromatic π–π stacking occurs in the crystal [minimum centroid–centroid separation = 3.7771 (15) Å].

## Related literature   

For background to Schiff bases derived from 2-hy­droxy-1-aromatic aldehydes and amines, see: Deneva *et al.* (2013 ▶); Martınez *et al.* (2011 ▶). For related structures, see: Albayrak *et al.* (2010 ▶); Petek *et al.* (2007 ▶). For reference bond lengths, see: Allen *et al.* (1987 ▶).
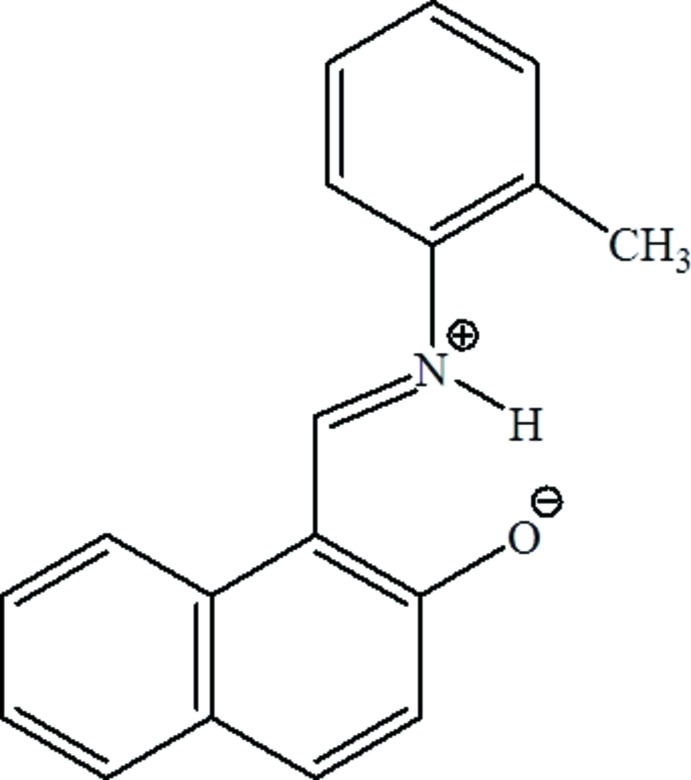


## Experimental   

### 

#### Crystal data  

C_18_H_15_NO
*M* *_r_* = 261.31
Orthorhombic, 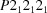

*a* = 7.3627 (5) Å
*b* = 12.4007 (10) Å
*c* = 14.4365 (12) Å
*V* = 1318.09 (18) Å^3^
*Z* = 4
Mo *K*α radiation
μ = 0.08 mm^−1^
*T* = 150 K
0.57 × 0.08 × 0.06 mm

#### Data collection  

Bruker APEXII CCD diffractometer
Absorption correction: multi-scan (*SADABS*; Bruker, 2006 ▶) *T*_min_ = 0.821, *T*_max_ = 0.995
14320 measured reflections
1721 independent reflections
1439 reflections with *I* > 2σ(*I*)
*R*_int_ = 0.046

#### Refinement  

*R*[*F* ^2^ > 2σ(*F* ^2^)] = 0.041
*wR*(*F* ^2^) = 0.111
*S* = 1.13
1721 reflections
182 parameters
H-atom parameters constrained
Δρ_max_ = 0.26 e Å^−3^
Δρ_min_ = −0.19 e Å^−3^

### 

Data collection: *APEX2* (Bruker, 2006 ▶); cell refinement: *SAINT* (Bruker, 2006 ▶); data reduction: *SAINT*; program(s) used to solve structure: *SIR97* (Altomare *et al.*, 1999 ▶); program(s) used to refine structure: *SHELXL97* (Sheldrick, 2008 ▶); molecular graphics: *ORTEP-3 for Windows* (Farrugia, 2012 ▶); software used to prepare material for publication: *WinGX* (Farrugia, 2012 ▶).

## Supplementary Material

Crystal structure: contains datablock(s) I. DOI: 10.1107/S1600536814008794/hb7171sup1.cif

Structure factors: contains datablock(s) I. DOI: 10.1107/S1600536814008794/hb7171Isup2.hkl

CCDC reference: 998118

Additional supporting information:  crystallographic information; 3D view; checkCIF report

## Figures and Tables

**Table 1:** Hydrogen-bond geometry (Å, °)

*D*—H⋯*A*	*D*—H	H⋯*A*	*D*⋯*A*	*D*—H⋯*A*
N13—H13⋯O1	0.88	1.85	2.546 (3)	134

**Fig. 1.**
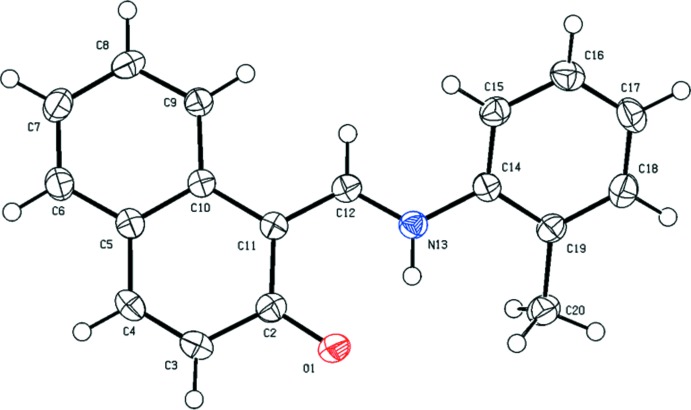
Molecular structure of the title compound with displacement ellipsoids drawn at the 50% probability level.

## supplementary crystallographic information

### 1. Comment

Schiff bases formed by condensation reactions of 2-hydroxy-1-aromatic aldehydes with various amines have been extensively studied (Deneva *et al.*, 2013; Martınez *et al.*, 2011). An interesting feature of these compounds is their faculty to display two possible tautomeric forms, the phenol-imine (OH) and the keto-amine (NH) forms. Depending on the tautomers, two types of intramolecular hydrogen bonds are observed in Schiff bases, O–H···N in phenol-imine and N–H···O in keto-amine forms. Another intermediate form of the Schiff base compounds is also known as zwitterion with an ionic intramolecular hydrogen bond N^+^–H···O^-^.

The molecular structure of (I) is illustrated in Fig. 1. The dihedral angle between the benzene ring and naphthalene ring is 33.7 (3)°. An intramolecular N—H···O hydrogen bond is found (Table 1).

The C12–N13 bond 1.312 (3) Å and the C2–O1 bond 1.301 (3) Å of the title compound are the most important indicators of the tautomeric type. While the C2–O1 bond is a double bond for a keto-amine tautomer, this bond has a single bond character in the corresponding phenol-imine tautomer; in addition, the C12–N13 bond is also a double bond in the phenol-imine tautomer but is a single bond length in the keto–amine tautomer (Albayrak *et al.*, 2010; Petek *et al.*, 2007). However, in the title Schiff base, these bond distances have intermediate values between single and double bonds which are 1.362 Å and 1.222 Å respectively for C–O and 1.339 and 1.279 Å respectively for C–N bond distance (Allen *et al.*, 1987). The shortened C2–O1 bond and the slightly longer C12–N13 bond provide structural evidence for the zwitterionic tautomeric form of the title compound.

### 2. Experimental

A mixture of a solution containing (3 mmol) of 2-hydroxy-1-naphthaldehyde and (3 mmol) of *o*-toluidine in 8 ml absolute ethanol. The mixture was stirred and heated under reflux for *ca* 5 h. The resulting solution was reduced under vacuum and cooled. A yellow solid was obtained; filtered off, washed with cold water and dried, the product was recrystallized from acetonitrile solvent as yellow rods.

### 3. Refinement

All non-hydrogen atoms were refined with anisotropic atomic displacement parameters. All H atoms, attached to carbon atoms have been placed in calculated positions positions and refined as riding, with C—H = 0.95 (aromatic), 0.98 Å(methyl) and N—H = 0.88, respectively, and *U*_iso_(H) = 1.2 *U*_eq_(C,*N*) or 1.5 *U*_eq_ (C_methyl_). The absolute structure was indeterminate in the present experiment.

### Crystal data

**Table:**

C_18_H_15_NO	*F*(000) = 552
*M**_r_* = 261.31	*D*_x_ = 1.317 Mg m^−^^3^
Orthorhombic, *P*2_1_2_1_2_1_	Mo *K*α radiation, λ = 0.71073 Å
Hall symbol: P 2ac 2ab	Cell parameters from 3889 reflections
*a* = 7.3627 (5) Å	θ = 2.8–26.6°
*b* = 12.4007 (10) Å	µ = 0.08 mm^−^^1^
*c* = 14.4365 (12) Å	*T* = 150 K
*V* = 1318.09 (18) Å^3^	Rod, yellow
*Z* = 4	0.57 × 0.08 × 0.06 mm

### Data collection

**Table:**

Bruker APEXII CCD diffractometer	1439 reflections with *I* > 2σ(*I*)
Graphite monochromator	*R*_int_ = 0.046
CCD rotation images, thin slices scans	θ_max_ = 27.5°, θ_min_ = 3.1°
Absorption correction: multi-scan (*SADABS*; Bruker, 2006)	*h* = −9→9
*T*_min_ = 0.821, *T*_max_ = 0.995	*k* = −16→16
14320 measured reflections	*l* = −18→18
1721 independent reflections	

### Refinement

**Table:**

Refinement on *F*^2^	Primary atom site location: structure-invariant direct methods
Least-squares matrix: full	Secondary atom site location: difference Fourier map
*R*[*F*^2^ > 2σ(*F*^2^)] = 0.041	Hydrogen site location: inferred from neighbouring sites
*wR*(*F*^2^) = 0.111	H-atom parameters constrained
*S* = 1.13	*w* = 1/[σ^2^(*F*_o_^2^) + (0.0543*P*)^2^ + 0.2897*P*] where *P* = (*F*_o_^2^ + 2*F*_c_^2^)/3
1721 reflections	(Δ/σ)_max_ = 0.004
182 parameters	Δρ_max_ = 0.26 e Å^−^^3^
0 restraints	Δρ_min_ = −0.19 e Å^−^^3^

### Special details

**Table:**

Geometry. All e.s.d.'s (except the e.s.d. in the dihedral angle between two l.s. planes) are estimated using the full covariance matrix. The cell e.s.d.'s are taken into account individually in the estimation of e.s.d.'s in distances, angles and torsion angles; correlations between e.s.d.'s in cell parameters are only used when they are defined by crystal symmetry. An approximate (isotropic) treatment of cell e.s.d.'s is used for estimating e.s.d.'s involving l.s. planes.
Refinement. Refinement of *F*^2^ against ALL reflections. The weighted *R*-factor *wR* and goodness of fit *S* are based on *F*^2^, conventional *R*-factors *R* are based on *F*, with *F* set to zero for negative *F*^2^. The threshold expression of *F*^2^ > σ(*F*^2^) is used only for calculating *R*-factors(gt) *etc*. and is not relevant to the choice of reflections for refinement. *R*-factors based on *F*^2^ are statistically about twice as large as those based on *F*, and *R*- factors based on ALL data will be even larger.

### Fractional atomic coordinates and isotropic or equivalent isotropic displacement parameters (Å^2^)

**Table:**

	*x*	*y*	*z*	*U*_iso_*/*U*_eq_	
O1	0.9691 (3)	0.33259 (13)	0.36389 (11)	0.0330 (4)	
C2	0.9303 (3)	0.23027 (19)	0.36261 (17)	0.0263 (5)	
C3	0.9485 (3)	0.1707 (2)	0.27755 (17)	0.0285 (5)	
H3	0.9881	0.2069	0.2232	0.034*	
C4	0.9106 (3)	0.06523 (19)	0.27381 (16)	0.0278 (5)	
H4	0.925	0.0282	0.2167	0.033*	
C5	0.8489 (3)	0.00629 (18)	0.35345 (16)	0.0241 (5)	
C6	0.8096 (4)	−0.10430 (19)	0.34660 (18)	0.0312 (6)	
H6	0.8193	−0.1391	0.288	0.037*	
C7	0.7575 (4)	−0.16291 (19)	0.42255 (17)	0.0338 (6)	
H7	0.734	−0.238	0.4173	0.041*	
C8	0.7395 (4)	−0.11076 (18)	0.50764 (17)	0.0301 (6)	
H8	0.7034	−0.151	0.5605	0.036*	
C9	0.7730 (3)	−0.00172 (18)	0.51646 (16)	0.0251 (5)	
H9	0.7577	0.0322	0.5749	0.03*	
C10	0.8301 (3)	0.06008 (17)	0.43913 (15)	0.0215 (5)	
C11	0.8716 (3)	0.17464 (18)	0.44429 (16)	0.0220 (5)	
C12	0.8560 (3)	0.23193 (18)	0.52831 (16)	0.0234 (5)	
H12	0.8199	0.1937	0.5823	0.028*	
N13	0.8891 (3)	0.33555 (15)	0.53540 (13)	0.0240 (4)	
H13	0.9142	0.3715	0.4844	0.029*	
C14	0.8867 (3)	0.39324 (17)	0.62052 (15)	0.0231 (5)	
C15	0.9417 (3)	0.34405 (19)	0.70234 (17)	0.0274 (5)	
H15	0.98	0.2709	0.7018	0.033*	
C16	0.9410 (4)	0.4015 (2)	0.78481 (18)	0.0317 (6)	
H16	0.9789	0.368	0.8407	0.038*	
C17	0.8846 (4)	0.5081 (2)	0.78508 (18)	0.0331 (6)	
H17	0.8822	0.5475	0.8415	0.04*	
C18	0.8319 (4)	0.55710 (19)	0.70344 (18)	0.0313 (6)	
H18	0.7946	0.6304	0.7046	0.038*	
C19	0.8321 (3)	0.50159 (18)	0.61949 (16)	0.0257 (5)	
C20	0.7722 (4)	0.55641 (19)	0.53136 (18)	0.0325 (6)	
H20A	0.8649	0.5461	0.4834	0.049*	
H20B	0.7558	0.6337	0.5429	0.049*	
H20C	0.6571	0.5251	0.5105	0.049*	

### Atomic displacement parameters (Å^2^)

**Table:**

	*U*^11^	*U*^22^	*U*^33^	*U*^12^	*U*^13^	*U*^23^
O1	0.0429 (11)	0.0269 (8)	0.0292 (9)	−0.0071 (8)	−0.0015 (8)	0.0044 (7)
C2	0.0240 (13)	0.0271 (11)	0.0278 (12)	−0.0004 (10)	−0.0033 (10)	0.0019 (10)
C3	0.0268 (13)	0.0369 (13)	0.0218 (11)	−0.0019 (11)	0.0004 (10)	0.0019 (10)
C4	0.0273 (13)	0.0356 (13)	0.0206 (11)	0.0033 (10)	−0.0018 (10)	−0.0038 (9)
C5	0.0204 (12)	0.0269 (11)	0.0251 (11)	0.0040 (10)	−0.0032 (9)	−0.0015 (9)
C6	0.0359 (15)	0.0294 (12)	0.0284 (12)	0.0050 (11)	−0.0046 (11)	−0.0042 (10)
C7	0.0405 (16)	0.0231 (11)	0.0378 (14)	−0.0012 (12)	−0.0053 (12)	−0.0001 (10)
C8	0.0313 (14)	0.0263 (11)	0.0328 (13)	−0.0032 (10)	−0.0020 (11)	0.0078 (10)
C9	0.0258 (12)	0.0248 (10)	0.0247 (11)	0.0008 (10)	−0.0004 (10)	0.0003 (9)
C10	0.0174 (12)	0.0234 (10)	0.0236 (11)	0.0021 (9)	−0.0014 (9)	0.0001 (9)
C11	0.0193 (11)	0.0240 (10)	0.0227 (11)	0.0010 (9)	−0.0007 (9)	0.0003 (9)
C12	0.0188 (11)	0.0252 (10)	0.0261 (12)	0.0003 (9)	0.0026 (9)	0.0018 (9)
N13	0.0240 (10)	0.0243 (9)	0.0239 (10)	−0.0020 (8)	0.0003 (8)	0.0011 (8)
C14	0.0191 (11)	0.0262 (11)	0.0239 (12)	−0.0041 (9)	0.0030 (10)	−0.0018 (9)
C15	0.0263 (12)	0.0264 (11)	0.0295 (12)	−0.0035 (10)	−0.0016 (10)	0.0024 (10)
C16	0.0307 (14)	0.0379 (13)	0.0266 (12)	−0.0086 (11)	−0.0042 (11)	0.0023 (10)
C17	0.0348 (14)	0.0380 (13)	0.0266 (12)	−0.0087 (11)	0.0018 (11)	−0.0082 (11)
C18	0.0311 (14)	0.0263 (11)	0.0367 (13)	−0.0020 (10)	0.0026 (12)	−0.0032 (10)
C19	0.0246 (12)	0.0247 (11)	0.0278 (12)	−0.0036 (10)	0.0015 (10)	0.0015 (9)
C20	0.0359 (15)	0.0278 (11)	0.0338 (13)	0.0017 (11)	−0.0001 (12)	0.0042 (10)

### Geometric parameters (Å, º)

**Table:**

O1—C2	1.301 (3)	C12—N13	1.312 (3)
C2—C11	1.433 (3)	C12—H12	0.95
C2—C3	1.439 (3)	N13—C14	1.422 (3)
C3—C4	1.339 (3)	N13—H13	0.88
C3—H3	0.95	C14—C15	1.390 (3)
C4—C5	1.436 (3)	C14—C19	1.403 (3)
C4—H4	0.95	C15—C16	1.387 (3)
C5—C6	1.405 (3)	C15—H15	0.95
C5—C10	1.412 (3)	C16—C17	1.385 (3)
C6—C7	1.370 (3)	C16—H16	0.95
C6—H6	0.95	C17—C18	1.382 (4)
C7—C8	1.395 (3)	C17—H17	0.95
C7—H7	0.95	C18—C19	1.394 (3)
C8—C9	1.380 (3)	C18—H18	0.95
C8—H8	0.95	C19—C20	1.508 (3)
C9—C10	1.418 (3)	C20—H20A	0.98
C9—H9	0.95	C20—H20B	0.98
C10—C11	1.455 (3)	C20—H20C	0.98
C11—C12	1.410 (3)		
			
O1—C2—C11	121.6 (2)	N13—C12—C11	123.1 (2)
O1—C2—C3	119.5 (2)	N13—C12—H12	118.5
C11—C2—C3	118.9 (2)	C11—C12—H12	118.5
C4—C3—C2	121.1 (2)	C12—N13—C14	123.91 (19)
C4—C3—H3	119.5	C12—N13—H13	118
C2—C3—H3	119.5	C14—N13—H13	118
C3—C4—C5	122.1 (2)	C15—C14—C19	120.9 (2)
C3—C4—H4	119	C15—C14—N13	120.7 (2)
C5—C4—H4	119	C19—C14—N13	118.4 (2)
C6—C5—C10	120.2 (2)	C16—C15—C14	120.2 (2)
C6—C5—C4	120.4 (2)	C16—C15—H15	119.9
C10—C5—C4	119.5 (2)	C14—C15—H15	119.9
C7—C6—C5	121.3 (2)	C17—C16—C15	119.6 (2)
C7—C6—H6	119.4	C17—C16—H16	120.2
C5—C6—H6	119.4	C15—C16—H16	120.2
C6—C7—C8	119.0 (2)	C18—C17—C16	120.1 (2)
C6—C7—H7	120.5	C18—C17—H17	120
C8—C7—H7	120.5	C16—C17—H17	120
C9—C8—C7	121.2 (2)	C17—C18—C19	121.6 (2)
C9—C8—H8	119.4	C17—C18—H18	119.2
C7—C8—H8	119.4	C19—C18—H18	119.2
C8—C9—C10	120.7 (2)	C18—C19—C14	117.7 (2)
C8—C9—H9	119.7	C18—C19—C20	120.7 (2)
C10—C9—H9	119.7	C14—C19—C20	121.6 (2)
C5—C10—C9	117.6 (2)	C19—C20—H20A	109.5
C5—C10—C11	119.1 (2)	C19—C20—H20B	109.5
C9—C10—C11	123.3 (2)	H20A—C20—H20B	109.5
C12—C11—C2	119.3 (2)	C19—C20—H20C	109.5
C12—C11—C10	121.2 (2)	H20A—C20—H20C	109.5
C2—C11—C10	119.4 (2)	H20B—C20—H20C	109.5
			
O1—C2—C3—C4	179.7 (2)	C5—C10—C11—C12	−179.6 (2)
C11—C2—C3—C4	0.4 (4)	C9—C10—C11—C12	−0.4 (3)
C2—C3—C4—C5	0.5 (4)	C5—C10—C11—C2	0.0 (3)
C3—C4—C5—C6	179.7 (2)	C9—C10—C11—C2	179.3 (2)
C3—C4—C5—C10	−1.1 (4)	C2—C11—C12—N13	1.6 (3)
C10—C5—C6—C7	−1.8 (4)	C10—C11—C12—N13	−178.8 (2)
C4—C5—C6—C7	177.4 (2)	C11—C12—N13—C14	−175.4 (2)
C5—C6—C7—C8	1.5 (4)	C12—N13—C14—C15	33.7 (3)
C6—C7—C8—C9	0.0 (4)	C12—N13—C14—C19	−148.0 (2)
C7—C8—C9—C10	−1.1 (4)	C19—C14—C15—C16	0.9 (4)
C6—C5—C10—C9	0.7 (3)	N13—C14—C15—C16	179.2 (2)
C4—C5—C10—C9	−178.5 (2)	C14—C15—C16—C17	0.1 (4)
C6—C5—C10—C11	−180.0 (2)	C15—C16—C17—C18	−0.9 (4)
C4—C5—C10—C11	0.8 (3)	C16—C17—C18—C19	0.6 (4)
C8—C9—C10—C5	0.7 (3)	C17—C18—C19—C14	0.4 (4)
C8—C9—C10—C11	−178.6 (2)	C17—C18—C19—C20	179.4 (3)
O1—C2—C11—C12	−0.2 (4)	C15—C14—C19—C18	−1.2 (3)
C3—C2—C11—C12	179.0 (2)	N13—C14—C19—C18	−179.5 (2)
O1—C2—C11—C10	−179.9 (2)	C15—C14—C19—C20	179.9 (2)
C3—C2—C11—C10	−0.6 (3)	N13—C14—C19—C20	1.5 (3)

### Hydrogen-bond geometry (Å, º)

**Table:**

*D*—H···*A*	*D*—H	H···*A*	*D*···*A*	*D*—H···*A*
N13—H13···O1	0.88	1.85	2.546 (3)	134
